# Discrete functions of GSK3α and GSK3β isoforms in prostate tumor growth and micrometastasis

**DOI:** 10.18632/oncotarget.3335

**Published:** 2015-01-21

**Authors:** Fei Gao, Ahmad Al-Azayzih, Payaningal R. Somanath

**Affiliations:** ^1^ Clinical and Experimental Therapeutics, College of Pharmacy, University of Georgia and Charlie Norwood VA Medical Center, Augusta, GA, USA; ^2^ College of Pharmacy, Jordan University of Science and Technology, Irbid, Jordan; ^3^ Department of Medicine, Vascular Biology Center and Cancer Center, Georgia Regents University, Augusta, GA, USA

**Keywords:** GSK3α, GSK3β, invasion, micrometastasis, prostate cancer

## Abstract

Isoform specific function of glycogen synthase kinase-3 (GSK3) in cancer is not well defined. We report that silencing of GSK3α, but not GSK3β expression inhibited proliferation, survival and colony formation by the PC3, DU145 and LNCaP prostate cancer cells, and the growth of PC3 tumor xenografts in athymic nude mice. Silencing of GSK3α, but not GSK3β resulted in reduced proliferation and enhanced apoptosis in tumor xenografts. ShRNA-mediated knockdown of GSK3α and GSK3β equally inhibited the ability of prostate cancer cells to migrate and invade the endothelial-barrier *in vitro*, and PC3 cell micrometastasis to lungs *in vivo*. Mechanistically, whereas silencing GSK3α resulted in increased expression of pro-apoptotic markers cleaved caspase-3 and cleaved caspase-9 in LNCaP, PC3 and DU145 cells, silencing GSK3β resulted in the inhibition of cell scattering, establishment of cell-cell contacts, increased expression and membrane localization of β-catenin, and reduced expression of epithelial to mesenchymal transition (EMT) markers such as Snail and MMP-9. This indicated the specific role of GSK3β in EMT, acquisition of motility and invasive potential. Overall, our data demonstrated the isoform specific role of GSK3α and GSK3β in prostate cancer cells *in vitro*, and tumor growth and micrometastasis *in vivo*, via distinct molecular and cellular mechanisms.

## INTRODUCTION

Although glycogen synthase kinase-3 (GSK3) has assumed a very unique place in various signaling pathways, their precise role in various cellular processes still remains unclear. The activity of GSK3 is negatively regulated by protein kinase B also known as Akt [1, 2]. Akt has been implicated in oncogenic transformation, tumor growth and metastasis of various cancers [3, 4]. Since GSK3 activity is inhibited by Akt-mediated phosphorylation at Serine 21 and Serine 9 in two different isoforms namely GSK3α and GSK3β respectively [5, 6], scientists believed that activation of GSK3 may likely suppress cancer progression. On the contrary, recent reports indicated that inhibition of GSK3 activity has tumor suppressive effect on various cancers [7-12], raising the question how Akt and GSK3 can concurrently be active in cancers. We recently provided the first evidence that both Akt and GSK3 can be maintained active simultaneously in prostate cancer cells and mouse embryonic fibroblasts via another activating phosphorylation of a tyrosine residue in GSK3 (Tyrosine 216) by Src family of kinases [13]. However, until today precise role of GSK3 in multiple cellular functions and clinical conditions is controversial, and isoform specific functions of GSK3 and their specific downstream targets in various cancers remain unclear.

Isoforms of GSK3 are identical in their kinase domain morphology (98% homology) and *in vitro* substrate specificities [14]. GSK3α structurally differs from GSK3β by possessing a glycine-rich extension in the N-terminal region and just 36% homology in the C-terminal region [15, 16]. Whereas GSK3β is the most studied and better characterized GSK3 isoform for its predominant expression in a majority of the cells and tissues [2], and for its specific involvement in the Wnt signaling cascade [17], specific function of GSK3α is less known. The presence of a glycine rich extension in the N-terminal region and variations in the C-terminal region in GSK3α suggests its recruitment to protein complexes different from that of GSK3β. The fact that GSK3α knockout mice are viable [18] and GSK3β knockout mice is embryonically lethal [19] further supports the hypothesis that GSK3 isoforms are not functionally redundant. While GSK3β is ubiquitously expressed, until today, the only cells known to express GSK3α predominantly as compared to GSK3β are spermatozoa [20]. This decade old study from our laboratory established a link between increased activation and reduced phosphorylation of GSK3α at serine 21 with increased sperm motility. Since then, there have been no reports indicating the predominant expression of GSK3α over GSK3β in any tissues, and most of the studies until today were focused on the GSK3β isoform.

Majority of the conclusions on the inhibitory role of GSK3 on various cellular functions came from mere correlative studies based on the assumption that serine phosphorylated GSK3 is functionally inactive. However, several recent studies, including ours indicated that GSK3 inhibition directly impairs the cancer cell function *in vitro,* and growth and metastasis of multiple cancers *in vivo* such as prostate [13], pancreas [9, 10], oral [8], and ovarian [21]. Reports indicated the specific role of GSK3β isoform in pancreatic [7] and non-small cell lung cancer cells [22]. Recently, an elegant study from Albert Baldwin group demonstrated for the first time that GSK3α plays a predominant role in pancreatic cancer, as compared to GSK3β [10]. In this report, GSK3α promoted oncogenic K-Ras function in pancreatic cancer cells through stabilization of TGFβ activated kinase-1 (TAK1) and TAK1 binding partner (TAB) interactions and subsequent NFκB activation [10, 23], suggesting that both these isoforms may have unique roles in various cancers. This advocates that irrespective of its expression levels, GSK3α, in addition to GSK3β should be taken into confidence while targeting GSK3 for cancer therapy.

Phosphorylation of the androgen receptor (AR) hinge and ligand-binding sites by GSK3β has been reported to inhibit expression of AR gene targets, thus inhibiting androgen-dependent prostate cancer cell proliferation [24, 25]. In contrast, GSK3β also have been implicated in AR gene expression [26]. Another study indicated that ShRNA-mediated knockdown and pharmacological inhibition of GSK3 inhibited AR expression and its transcriptional activity in prostate cancer cells [27]. These reports were very inconclusive to inform us whether it would be beneficial or harmful to target GSK3 for androgen-dependent prostate cancer. We reported the first evidence on the role of GSK3 in advanced, androgen insensitive prostate cancer cells. Pharmacological inhibition or SiRNA-mediated knockdown of GSK3 inhibited androgen-independent prostate cancer cell function *in vitro* and tumor growth *in vivo* [13]. This was in agreement with the clinical report from human prostate cancer patient tumor tissues indicating increased protein and mRNA expression of GSK3α starting from the early tumor growth and increased expression of GSK3β specifically in advanced cancers [28]. This suggested distinct roles for GSK3α and GSK3β in the early and later stages of prostate cancer growth. Interestingly, expressions of both GSK3 isoforms were elevated in advanced prostate cancer tissues further indicating that GSK3α may also be needed in advanced prostate cancer.

In the current study, we provide the first evidence that the regulation of cell survival, proliferation and rate of tumor growth in early (LNCaP) and advanced prostate cancer (PC3 and DU145) cells are predominantly dependent on GSK3α. In contrast, the promotion of epithelial to mesenchymal transition (EMT) and acquisition of invasive and metastatic property in advanced prostate cancer cells is more dependent on GSK3β-mediated inhibition of β-catenin expression and destabilization of cell-cell contacts. Since knocking down GSK3α in prostate cancer cells is much more effective in inhibiting prostate tumor growth and colonization compared to GSK3β, our study reveal that inhibition of GSK3α or even better, pharmacological inhibition of both GSK3 isoforms will be an effective strategy for prostate cancer therapy.

## RESULTS

### Silencing GSK3α gene inhibits prostate cancer cell proliferation

**Figure 1:**
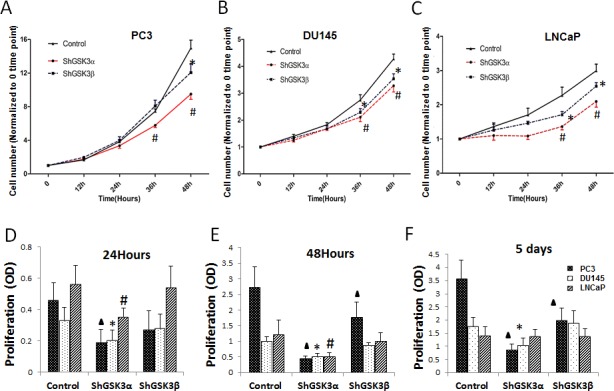
GSK3α regulates prostate cancer cell proliferation (A) Figure showing PC3 cells stable transfected with ShRNAs for scrambled, GSK3α and GSK3β via lentiviral infections followed by antibiotic selection plated in 12 well plates and counted at 0, 12, 24, 36 and 48 hours (n=4). (B) Figure showing DU-145 cells stable transfected with ShRNAs for scrambled, GSK3α and GSK3β plated in 12 well plates and counted at 0, 12, 24, 36 and 48 hours (n=4). (C) Figure showing LNCaP cells stable transfected with ShRNAs for scrambled, GSK3α and GSK3β plated in 12 well plates and counted at 0, 12, 24, 36 and 48 hours (n=4). (D) Bar graph showing PC3 cells stable transfected with ShRNAs for scrambled, GSK3α and GSK3β subjected for MTT assay demonstrating the effect of ShRNA-mediated knockdown of GSK3α and GSK3β on cell proliferation at 12, 24 and 48 hours (n=8). (E) Bar graph showing DU145 cells stable transfected with ShRNAs for scrambled, GSK3α and GSK3β subjected for MTT assay demonstrating the effect of ShRNA-mediated knockdown of GSK3α and GSK3β on cell proliferation (n=8). (F) Bar graph showing LNCaP cells stable transfected with ShRNAs for scrambled, GSK3α and GSK3β subjected for MTT assay demonstrating the effect of ShRNA-mediated knockdown of GSK3α and GSK3β on cell proliferation (n=8). Data is shown as Mean + SD; ^▲,*,#^p<0.05 for PC3, DU145 and LNCaP cells, respectively.

In order to determine the isoform specific role of GSK3 in the regulation of prostate cancer cell growth and proliferation, we generated PC3, DU145 and LNCaP stable cell lines expressing ShRNAs for control (non-target), GSK3α and GSK3β (ShControl, ShGSK3α and ShGSK3β, respectively). In our analysis, GSK3α deficient PC3, DU145 and LNCaP cells exhibited reduced cell growth as compared to control, as measured at 36 and 48 hours post plating of equal number of cells in the wells. Although a significant inhibition of cell growth was also observed in GSK3β deficient PC3, DU145 and LNCaP cells, effect was modest when compared to the GSK3α deficient cells (Figure 1A-C). Our further analysis with MTT confirmed that absence of GSK3α expression in PC3, DU145 and LNCaP cells significantly inhibit their proliferation (Figure 1D-F). Our results indicated that prostate cancer cell proliferation is predominantly regulated by GSK3α isoform.

### GSK3α is necessary for prostate cancer colony formation *in vitro* and well as proliferation and tumor growth *in vivo*

**Figure 2:**
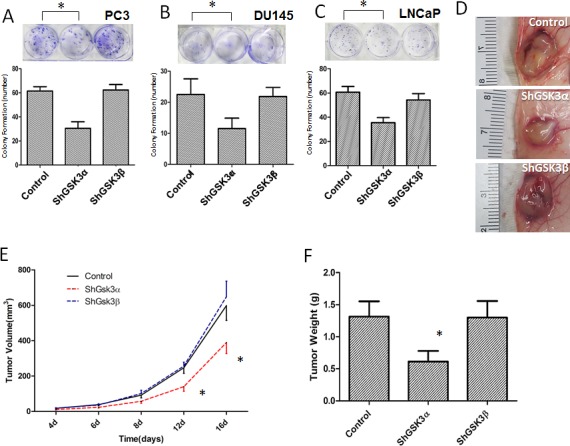
GSK3α, but not GSK3β is necessary for prostate cancer cell colony formation *in vitro* and tumor growth *in vivo* (A) Figure (upper panel) and bar graph (lower panel) showing colony formation of PC3 cells with stable knockdown of GSK3α and GSK3β, compared to PC3 cells expressing control ShRNA (n=4). (B) Figure (upper panel) and bar graph (lower panel) showing colony formation of DU145 cells with stable knockdown of GSK3α and GSK3β, compared to PC3 cells expressing control ShRNA (n=4). (C) Figure (upper panel) and bar graph (lower panel) showing colony formation of DU145 cells with stable knockdown of GSK3α and GSK3β, compared to PC3 cells expressing control ShRNA (n=4). (D) Images of PC3 cell tumor xenografts collected from athymic nude mice with cells expressing control (upper), GSK3α (middle) and GSK3β (lower) ShRNAs. (E) Figure showing tumor volume of the PC3 xenografts in athymic nude mice expressing control, GSK3α and GSK3β ShRNAs on day 4, 6, 8, 12 and 16 after injection (n=6). (F) Bar graph showing tumor weight of the PC3 xenografts in athymic nude mice expressing control, GSK3α and GSK3β ShRNAs collected on day 16 after injection (n=6). Data is shown as Mean + SD; *p <0.05.

From our *in vitro* studies, we know that silencing GSK3α predominantly inhibits prostate cancer cell proliferation. In order to determine if this has any effect on the overall tumor growth, we subjected ShControl, ShGSK3α and ShGSK3β expressing PC3, DU145 and LNCaP cells for colony (foci) formation assay *in vitro* and PC3 cells for tumor xenograft growth *in vivo*. Our analysis indicated that knocking down GSK3α in PC3, DU145 or LNCaP cells resulted in reduced colony formation *in vitro* (Figure 2A-C). In contrast, PC3, DU145 and LNCaP cells deficient in GSK3β did not show any significant effect on colony formation compared to respective ShControl cells (Figure 2A-C). Similarly, we observed significantly reduced tumor growth in ShGSK3α PC3 cell tumor xenografts in athymic nude mice as compared to ShControl tumor xenografts (Figure 2D-F). However, no significant difference in the growth of ShGSK3β PC3 tumor xenografts was observed as compared to ShControl PC3 xenografts (Figure 2D-F).

**Figure 3:**
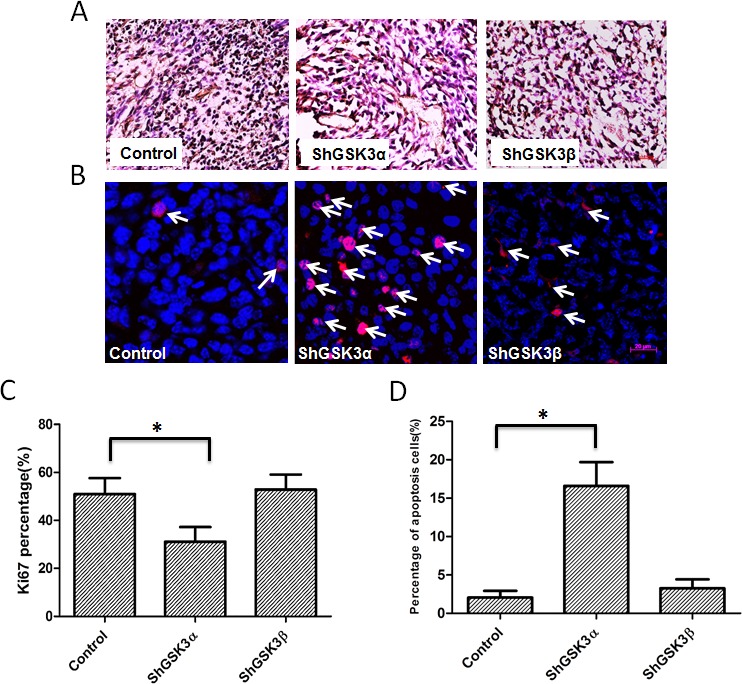
GSK3α, but not GSK3β regulates cell survival and proliferation of PC3 cells in athymic nude mice tumor xenografts (A) Microscopic images of control, GSK3α and GSK3β ShRNA expressing PC3 tumor xenograft sections subjected for ki67 staining showing proliferating cells. (B) Fluorescent microscopic images of control, GSK3α and GSK3β ShRNA expressing PC3 tumor xenograft sections subjected for TUNEL assay showing apoptotic cells. (C) Bar graph showing percentage of proliferating PC3 cells in tumor xenografts transfected with control, GSK3α and GSK3β ShRNA, normalized to the total number of cells (n=6). (D) Bar graph showing percentage of apoptotic cells in PC3 cells in tumor xenografts transfected with control, GSK3α and GSK3β ShRNA, normalized to the number of nuclei (n=6). Data is shown as Mean + SD; *p <0.05.

Next, we determined if GSK3α knockdown also affect tumor cell proliferation *in vivo*. To do this, we performed immunohistochemistry analysis of tumor xenograft frozen sections with proliferation marker Ki67. Our analysis indicated that ShGSK3α PC3 cell tumor xenografts exhibit reduced number of Ki67 positive (proliferating) cells as compared to ShControl tumor xonografts (Figure 3A and C). However, knocking down GSK3β in PC3 cells did not elicit any effect on the proliferation *in vivo* (Figure 3A and C). Using TUNEL staining, we also determined which GSK3 isoform is involved in the regulation of cell survival and apoptosis in prostate tumor xenografts. Our analysis showed that while knocking down GSK3α in PC3 cells increased the number of TUNEL positive (apoptotic) cells in tumors, knocking down GSK3β did not had any effect on apoptosis as compared to control tumors (Figure 3B and D). Thus, our results demonstrated that GSK3α, and not GSK3β is necessary for the prostate cancer cell proliferation, survival and colony formation *in vitro* and tumor xenograft growth *in vivo*.

### Silencing either GSK3α or GSK3β gene inhibits prostate cancer cell motility and microinvasion

**Figure 4:**
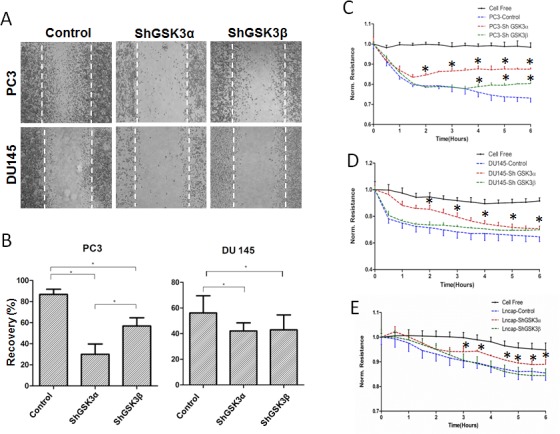
Reduced GSK3α or GSK3β expression leads to inhibition of prostate cancer cell motility and microinvasion *in vitro* (A) Microscopic images of PC3 (upper panel) and DU145 (lower panel) cell monolayer scratches expressing control, GSK3α and GSK3β ShRNA, 12 hours after creation of scratches showing the effect of GSK3α and GSK3β knockdown on prostate cancer cell motility. (B) Bar graph showing the ability of control, GSK3α and GSK3β ShRNA expressing PC3 and DU145 cells to migrate and recover the wound (by filling the scratch area) (n=6). (C) Figure showing the ability of control, GSK3α and GSK3β ShRNA expressing PC3 cells to migrate through the endothelial monolayer (microinvasion) as measured using the electric cell-substrate impedance sensing technology (n=4). (D) Figure showing the ability of control, GSK3α and GSK3β ShRNA expressing DU145 cells to migrate through the endothelial monolayer (n=4). (E) Figure showing the ability of control, GSK3α and GSK3β ShRNA expressing LNCaP cells to migrate through the endothelial monolayer (n=4). Data is shown as Mean + SD; *p <0.05.

Whereas proliferation and cell survival is necessary for the tumor growth, cell motility and invasive ability is crucial for metastasis. Hence, we next determined the effect of isoform specific knockdown of GSK3α and GSK3β in prostate cancer cells on their motility and transendothelial migration (microinvasion). The rate of cell motility was determined based on the conventional monolayer scratch recovery assay. For ECIS invasion assay *in vitro*, ShGSK3α and ShGSK3β PC3 and DU145 cells were introduced on top of human microvascular endothelial cell (HMEC) monolayer and the changes in electrical resistance offered by the cell-barrier were measured by the automated reader through the gold-plated electrodes connecting array chips with the ECIS equipment [13]. Our data indicated that silencing either GSK3α or GSK3β in PC3 or DU145 cells inhibited cell motility as evidenced by the impaired scratch recovery (Figure 4A and B), and impaired microinvasion by PC3, DU145 and LNCaP cells as measured by the increased HMEC-barrier resistance (Figure 4C-E). Interestingly, on both occasions, effect of GSK3α knockdown was predominant over GSK3β (Figure 4A-D). Together, these results indicated that both GSK3α and GSK3β isoforms are necessary for the prostate cancer cell motility and invasion.

### Silencing either GSK3α or GSK3β gene inhibits prostate cancer colonization in lungs

**Figure 5:**
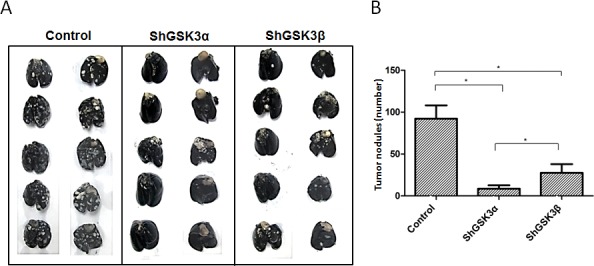
ShRNA-mediated silencing of both GSK3α and GSK3β gene expression inhibited prostate cancer colonization to lungs (A) Figure showing the lungs collected from athymic nude mice 16 days after intravenous (tain-vein) administration of PC3 cells expressing control, GSK3α and GSK3β ShRNA and perfused with india ink to visualize the metastatic tumor nodules. (B) Bar graph showing the number of tumor nodules quantified from the external surface of athymic nude mice lungs collected 16 days after intravenous administration of PC3 cells expressing control, GSK3α and GSK3β ShRNA showing the effect of GSK3α and GSK3β gene knockdown on their ability to metastasize to the lungs (n=5). Data is shown as Mean + SD; *p <0.05.

**Figure 6:**
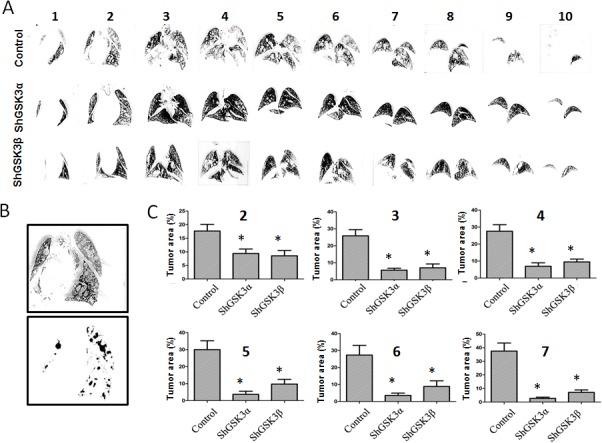
GSK3α gene silencing has improved therapeutic benefits as compared to GSK3β in inhibiting prostate cancer colonization to lungs (A) Figure showing serial sections of the lungs collected from athymic nude mice 16 days after intravenous (tain-vein) administration of PC3 cells expressing control, GSK3α and GSK3β ShRNA and perfused with india ink to visualize the metastatic tumor nodules. (B) Figure showing image conversion using Image J software to clearly visualize and quantify metastasized tumor nodules in the lungs. (C) Bar graph showing the area of tumor growth in each lung serial sections collected 16 days after intravenous administration of PC3 cells expressing control, GSK3α and GSK3β ShRNA showing the effect of GSK3α and GSK3β gene knockdown on their ability to metastasize to the lungs (n=5). Data is shown as Mean + SD; *p <0.01.

Next, we determined if silencing either of the GSK3 isoforms will have any effect on metastasis *in vivo*. To do this, we used a modified method of the previously developed lung micrometastasis model mice [29]. Our *in vivo* data on lung colonization in athymic nude mice of ShControl, ShGSK3α and ShGSK3β PC3 cells was highly in agreement with our *in vitro* data on the effect of GSK3 isoforms on cell motility and transendothelial migration. Whereas silencing of either ShGSK3α or ShGSK3β in PC3 cells impaired colonization to lungs in athymic nude mice compared to ShControl, the effect of silencing ShGSK3α was more effective than silencing ShGSK3β in PC3 cells (Figure 5A and B). In order to confirm this further, we subjected the mice lungs for serial section analysis to determine the extent of colonization of PC3 cells. Our analysis showed that both ShGSK3α and ShGSK3β gene knockdown resulted in impaired colonization of PC3 cells to the mice lungs (Figure 6A-C). Thus, our results demonstrate the importance of ShGSK3α and ShGSK3β isoforms in prostate cancer invasion and micrometastasis.

### GSK3α and GSK3β specifically mediate prostate cancer cell intrinsic survival pathway and epithelial-to-mesenchymal transition (EMT), respectively

**Figure 7:**
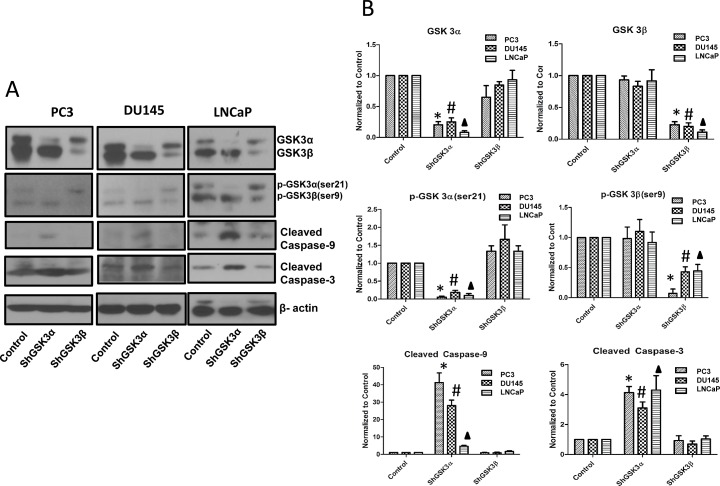
GSK3α, and not GSK3β regulates intrinsic caspase-3/9 dependent survival pathway in prostate cancer cells (A) Western blot images showing the effect of GSK3α and GSK3β gene silencing in PC3 (left panel), DU145 (center panel) and LNCaP (right panel) cells on the levels of cleaved caspase-9 and cleaved caspase-3, important mediators of intrinsic apoptotic pathway. (B) Densitometry analysis of Western blot images showing the effect of GSK3α and GSK3β gene silencing in PC3, DU145 and LNCaP cells on the levels of cleaved caspase-9 and cleaved caspase-3, along with phosphorylated and total levels of GSK3α and GSK3β (n=3). Data is shown as Mean + SD; ^*,#,▲^p<0.05 for PC3, DU145 and LNCaP cells, respectively.

Cell based assays indicated a predominant effect of GSK3α knockdown in prostate cancer cells on cell survival, proliferation and tumor growth with only a modest or no effect of GSK3β knockdown on these cell functions. However, the observed functional overlap between GSK3α and GSK3β gene silencing on cell motility, invasion and micrometastasis *in vivo* questioned if prostate cancer metastasis is a redundant function of both the GSK3 isoforms. To address this, we determined the effect of isoform specific knockdown of GSK3α and GSK3β on cellular pathways regulating cell survival as well as cell motility and invasion. Western analysis of ShControl, ShGSK3α and ShGSK3β revealed that silencing GSK3α, but not GSK3β results in increased expression of pro-apoptotic cleaved caspase-9 and cleaved caspase-3 in all the three prostate cancer cell lines (Figure 7A and B), thus indicating the GSK3α is directly involved in the intrinsic pro-survival pathway in prostate cancer cells.

**Figure 8:**
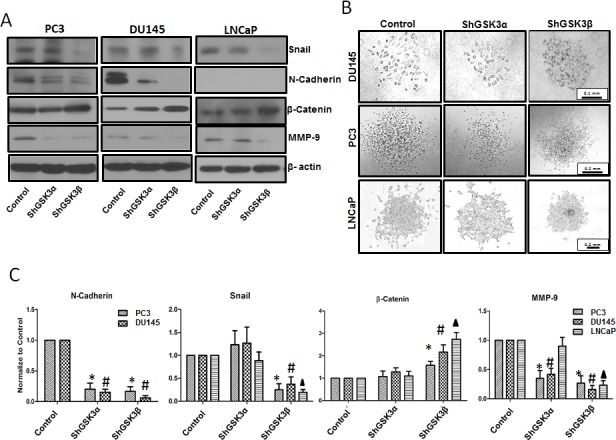
GSK3β predominantly regulates prostate cancer epithelial-to-mesenchymal transition via a Snail/N-Cadherin/β-Cateinin pathway (A) Western blot images showing the effect of GSK3α and GSK3β gene silencing in PC3 (left panel), DU145 (center panel) and LNCaP (right panel) cells on the expression of epithelial and mesenchymal specific genes Snail, N-Cadherin, β-catenin and MMP-9. (B) Microscopic images of PC3 (upper panel), DU145 (center panel) and LNCaP (lower panel) cells displaying phenotypic changes between cobblestone-like epithelial morphology and typical mesenchymal-like morphology with silencing of GSK3α and GSK3β gene expression as compared to control cells. (C) Densitometry analysis of Western blot images showing the effect of GSK3α and GSK3β gene silencing in PC3, DU145 and LNCaP cells on the expression of Snail, N-Cadherin, β-catenin and MMP-9 (n=3). Data is shown as Mean + SD; ^*,#,▲^p<0.05 for PC3, DU145 and LNCaP cells, respectively.

On the other end, cellular processes such as epithelial-to-mesenchymal transition (EMT), cell motility, transendothelial migration and invasion are essential for the cancer metastasis. Our Western analysis of ShControl, ShGSK3α and ShGSK3β PC3, DU145 and LNCaP cells revealed that silencing GSK3β, but not GSK3α results in decreased expression of pro-EMT gene Snail associated with increased expression Wnt signaling mediator βcatenin (Figure 8A and C). Interestingly, reduced expression of some of the mediators of EMT and invasion such as N-cadherin (not detected in LNCaP cells) and matrix metalloprotease-9 (MMP9) were noticed with silencing of either of the GSK3 isoforms (Figure 8A and C). Due to this overlap, we subjected prostate cancer cells for cell scattering assay to specifically determine the role of each GSK3 isoform in EMT and motility. Our analysis indicated that upon plating equal number of ShControl, ShGSK3α and ShGSK3β PC3, DU145 and LNCaP cells in comparable conditions, ShGSK3β cells, and not ShControl or ShGSK3α PC3, DU145 and LNCaP cells exhibited the tendency to interact and aggregate each other, a reversal of EMT-mediated cell scattering. In contrast, more cell death and hence reduced number of plated cells was observed with GSK3α gene silencing in PC3, DU145 and LNCaP cells. Thus, our study demonstrated that prostate cancer EMT, a pre-requisite for invasion and colonization is a predominant function of GSK3β isoform, and that the effect of GSK3α on prostate cancer colonization may be secondary due to the increased apoptosis and reduced proliferation of prostate cancer cells.

### GSK3β gene silencing increases expression and membrane localization of β-catenin

**Figure 9:**
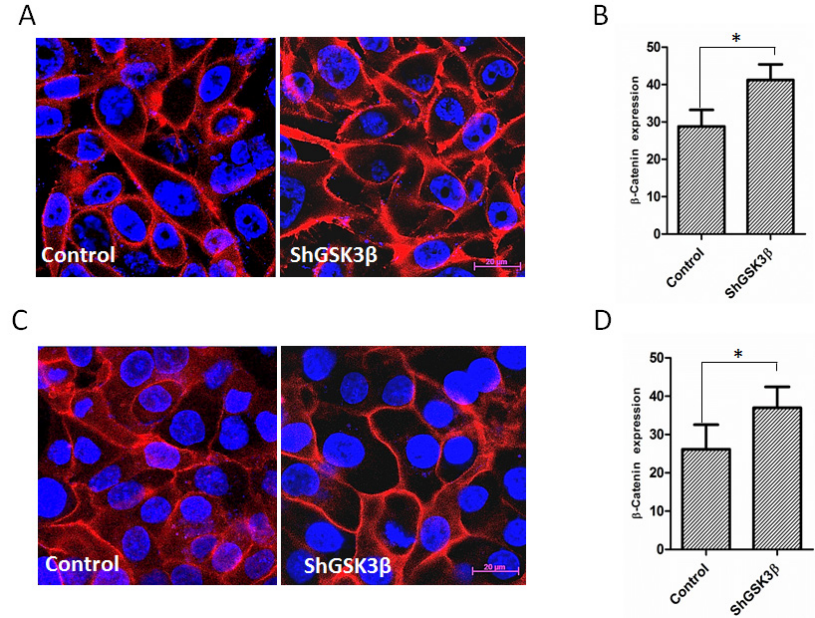
GSK3β gene silencing results in increased expression and membrane localization of β-catenin (A) Fluorescent images on ShControl and ShGSK3β PC3 cells probed with anti-β-catenin antibodies showing the intracellular localization of β-catenin in the presence of 10% FBS. (B) Quantification of the total expression of β-catenin in ShControl and ShGSK3β PC3 cells in optical density (OD) units as measured by the NIH Imgae J software. (C) Fluorescent images on ShControl and ShGSK3β DU145 cells probed with anti-β-catenin antibodies showing the intracellular localization of β-catenin in the presence of 10% FBS. (B) Quantification of the total expression of β-catenin in ShControl and ShGSK3β DU145 cells as measured in OD units by the NIH Imgae J software (n=9). Data is shown as Mean + SD; *p <0.01.

Since the effect of β-catenin on prostate cancer progression is more reliant on its intracellular localization in the nucleus, cytoplasm or on the membrane rather than its increased expression, we performed immunocytochemistry analysis of PC3 and DU145 prostate cancer cells. Our analysis indicated that in resting PC3 and DU145 cells, β-catenin is mostly present in the cytoplasm and the membrane with negligible amounts present in the nucleus (Figure 9A and C). Upon GSK3β gene silencing, expression of total β-catenin was significantly increased (Figure 9B and D). Interestingly, despite the increased β-catenin expression in ShGSK3β cells, its levels in the cytoplasm was reduced, and most of the β-catenin in the cells was localized on the membrane (Figure 9A and C), suggesting that GSK3β-mediated effect on the expression and localization of β-catenin is predominantly associated with barrier-junction signaling as compared to Wnt signaling.

## DISCUSSION

Whereas the impact of GSK3 activity modulation in multiple cancers by itself is a major reason for controversy and concern among scientists, their presence in two different isoforms, likely modulating various cellular processes in a non-redundant manner further complicated the problem. Hence, we investigated both these facets of the GSK3 conundrum in early stage LNCaP and advanced stage PC3 and DU145 human prostate cancer cell lines on cellular function, tumor growth and micrometastasis. In the current manuscript, we provide novel insights on the isoform specific role of GSK3α and GSK3β in prostate cancer cell proliferation, apoptosis, colony formation, motility and invasion *in vitro* as well as tumor growth and micrometastasis to lungs *in vivo* along with underlying molecular mechanisms.

Major focus of our laboratory is to understand the complex mechanisms by which Akt and its signaling partners regulate prostate cancer cell function *in vitro*, and tumor growth, angiogenesis and metastasis *in vivo*. In pursuit of this goal, we have so far shown that Akt1, predominant Akt isoform in prostate cancer cells is integral for tumor growth [30-32] and invasion [33]. The importance of PTEN-Akt pathway in cancer [34-37], including prostate cancer [38-41] have also been indicated by other laboratories. Interestingly, we have also demonstrated that Akt1 pathway can negatively regulate tumor angiogenesis and vascular permeability [42] and that Akt-independent mechanism also does exist in prostate cancer cells leading to cell survival [43]. These reports clearly indicate the need to characterize the downstream signaling pathways modulated by Akt in many cancers, not only to unveil the reasons for such discrepancies, but also to help designing ways to pharmacologically modulate Akt activity or target its downstream effectors for prostate cancer therapy.

One of the best known, but the least characterized substrate of Akt is the two gene family of GSK3. Akt phosphorylates Serine 21 and Serine 9 in GSK3α and GSK3β, respectively leading to their inactivation [5, 6]. Akt is a potent oncogene, and apart from inactivating mutations in PTEN, activating E17K mutations in Akt in prostate cancer cells have also demonstrated to promote prostate cancer [44]. Hence it was logical for the scientists to assume that prostate cancer cells may likely be more susceptible to pharmacological activation of GSK3 rather than its inhibition. Even though the earlier studies indicated occurrence of such a scenario in prostate cancer [24, 25], more recent studies indicated the opposite in multiple cancers [8-10, 13, 21, 22]. On one end, Wnt signaling triggered by Wnt ligand through frizzled is known to inhibit GSK3β thus stabilizing the β-catenin in the cytoplasm and its subsequent translocation to the nucleus [17]. Considering an oncogene, nuclear translocation of β-catenin is assumed to promote tumor growth via transcriptional activation. On the other end, β-catenin is also an integral component of the epithelial adherens junctions (AJ) [17], which is beyond its role in Wnt signaling. In association with epithelial cadherins (E-cadherin), β-catenin stabilizes cell-cell contacts, thus maintaining the epithelial cell polarity [45]. The latter although stabilizes the β-catenin expression in prostate cancer cells, it does so by anchoring it on the membrane in epithelial AJs, thus preventing its entry into the nucleus as indicated in clinical samples [46]. During EMT in prostate cancer, reduced expression of E-cadherin will release the β-catenin for translocation into the nucleus [47, 48] for pro-oncogenic function. Even down-regulation of β-catenin expression has also been reported in prostate cancer [49, 50]. Our results is a perfect documentation supporting the latter scenario, where knocking down GSK3β results in the increased expression of β-catenin, increased cell-cell contacts, re-establishing epithelial cell polarity and reduced cell scattering associated with reduced expression of EMT markers, collectively resulting in the inhibition of cell motility, invasion and micrometastasis *in vitro* and *in vivo*.

Our previous study reported that GSK3 activation as a result of Src-mediated phosphorylation of GSK3 tyrosine 216 is necessary for prostate cancer cell function *in vitro*. We also demonstrated that the inhibition of prostate cancer Src activity by treatment with Src-Abl inhibitor Dasatinib resulted in the inhibition of GSK3 tyrosine 216 phosphorylation in prostate cancer cell lines subsequently inhibiting proliferation, survival and invasion *in vitro* and resulting in impaired tumor growth *in vivo* [13]. A number of reports since mid-2000 have also revealed GSK3β as a prostate cancer promoter [2], further demonstrating the importance of GSK3β in prostate cancer. Despite these confirmations, two most recent reports emanating from the same laboratory argues the effect of GSK3β activation on EMT in prostate cancer [51, 52]. These studies claim that growth factors such as EGF and bFGF induce EMT in prostate cancer cells via PKC and Akt-mediated inhibition of GSK3β, respectively [51, 52] showing increased phosphorylation of its Serine 9 residue upon EGF or bFGF stimulation. One drawback in this study is that the authors went on the assumption that serine 9 phosphorylated GSK3β is functionally inactive, and activity not determined through an enzymatic assay or phosphorylation of β-catenin, a known GSK3β substrate. On the other end, clinical analysis performed in the prostate cancer patient samples indicated that while GSK3α and GSK3β expressions are highly elevated in the early and advanced stages of prostate cancer, respectively, expression of both the isoforms are significantly elevated in the advanced stages. This once again support our findings that GSK3α-mediated cell survival and proliferation promotes tumor growth in both early and advanced stages, and that GSK3β/β-catenin-mediated EMT promotes cell motility, invasion and micrometastasis in the advanced stages.

Intracellular β-catenin has multiple fates depending upon the stimuli and type of cells [17, 45]. Our data indicated that although expression of β-catenin was increased in ShGSK3β PC3 and DU145 cells compared to respective ShControl cells, its presence in the cytoplasm was reduced and its localization in the membrane was elevated. This indicated that in prostate cancer cells, GSK3β inhibition will lead to stabilization of cell-barrier inhibiting cell scattering and reversing EMT. Whereas most of the effect of GSK3α knockdown on prostate cancer micrometastasis may come from the effect of GSK3α deficiency on apoptosis and proliferation, a role for GSK3α in EMT and micrometastasis cannot be ruled out based on the effect of GSK3α gene knockdown on the expression of EMT markers such as N-cadherin and MMP9. Nevertheless, we provide the first report on the isoform specific function of GSK3α and GSK3β in the regulation of prostate cancer cell survival and proliferation, as well as EMT and micrometastasis, respectively. Thus, our study generates reasonable evidence to propose that targeting GSK3 can be an effective strategy to treat advanced stage prostate cancer.

## MATERIALS AND METHODS

### Animals

All animal procedures listed in this article were performed as per the protocol approved by the Institutional Animal Care and Use Committee at the Charlie Norwood Veterans Affairs Medical Center, Augusta, GA (ACORP# 09-07-011). All the efforts were made to minimize animal pain and suffering. Athymic nude mice necessary for the tumor xenograft and lung colonization experiments were purchased from Harlan Laboratories (Indianapolis, IN).

### Cell lines, reagents, and antibodies

Human PC3, DU145 and LNCaP cell lines were obtained from ATCC (Manassas, VA) and maintained in DMEM High Glucose (PC3 and DU145) or RPMI-1640 (LNCaP) (HyClone) with 10% fetal bovine serum (FBS), 100 units/ml penicillin, and 100 μg/ml streptomycin in 5% CO_2_ humidified atmosphere at 37°C. Primary antibodies against cleaved caspase-3, cleaved caspase-9, N-Cadherin, p-GSK3 Ser9/21, β-catenin and Snail were purchased from Cell Signaling (Boston, MA), and anti-β-actin was from Sigma (St Louis, MO). Anti-mouse and anti-rabbit HRP conjugated secondary antibodies were obtained from BioRad (Hercules, CA). Human dermal microvascular endothelial cells (HMEC) were obtained from ATCC. Cells were maintained in Endothelial Cell Basal Medium-2 (Lonza) with Microvascular Endothelial Cell Growth Medium-2 Bullet Kit (Lonza). All cultures were maintained in a humidified 5% CO2 incubator at 37°C, and routinely passaged when 80–90% confluent.

### Generation of ShGSK3α and ShGSK3β stable PC3, DU145 and LNCaP cell lines

Cells were infected with lenti-virus particles in 6 well plates. Medium was changed a day prior to lentivirus infection. PC3, DU145 and LNCaP (70-80 % confluent) were infected with SMART vector 2.0 lentivirus particles (10^9^ pfu) expressing ShGSK3α or ShGSK3β (containing a mixture of 3 ShRNA targeting GSK3α or GSK3β mRNA) or Non-targeting Control Particles (Dharmacon, Thermo Scientific). Lentivirus particles were mixed in 1ml SFM4Transfx-293 (Hyclone) and added along with 1 μl Polybrene (10mg/ml, American bioanalytical). PC3, DU145 and LNCaP were then incubated for 16 h, following which medium was changed to complete DMEM. After 3 days following lentivirus infection, transfection efficiency was tested by looking into the percentage of cells expressing Turbo-GFP using Fluorescence microscope. Prostate cancer cells with stable silencing of ShGSK3α or ShGSK3β gene (or stable expression of control non-coding ShRNA) were selected by treatment with 5 μg/ml puromycin (Invitrogen) treatment. Stable cell lines thus prepared were then cultured in complete DMEM medium with 0.4 μg/ml puromycin.

Electric cell-substrate impedance sensing (ECIS) assay for cancer cell microinvasion: Cancer cell transendothelial migration (microinvasion) was studied using the ability of cancer cells to penetrate the endothelial monolayer and thus disrupt the endothelial-barrier integrity (measured as electrical resistance of the endothelial monolayer) as determined using Electric Cell-substrate Impedance Sensing (ECIS) technology from Applied Biophysics (Troy, NY) [33, 53]. Briefly, HMEC were seeded in 1:1 density on gelatin-coated ECIS arrays (8W10E+), each containing 8 wells with 16 gold electrodes per well. Culture dishes were replenished with fresh medium 24 h after seeding and the experiments were started when the cells reach a monolayer. PC3, DU145 and LNCaP cells at a concentration of 0.5×10^5^/well were added on top of the HMEC monolayer. The resistance values were measured at multiple frequencies mode.

### Cell proliferation assay

Prostate cancer cell proliferation was studied as previously described [54]. Briefly, 200μL of medium containing 0.5 × 10^4^ PC3, DU145 and 1.0 × 10^4^ LNCaP cells were seeded in each well of 96-well plates. On the second day, medium were removed and a 200 ul of a new medium containing 0.5 mg/mL 3-(4,5-dimethylthiazole-2-yl)-2,5-diphenyltetrazolium bromide (MTT) were added to each well then incubated for 4 hours at 37 °C. Next, medium were removed, and 200 ul of isopropanol per well was added to solubilize the crystalized formazan products. Absorbance at 570 nm was measured using an automated microplate spectrophotometer (ELX800, Biotek).

### Cell migration assay

Cancer cell migration was performed as previously standardized [55]. Briefly, PC3 and DU145 cells expressing control, GSK3α or GSK3β ShRNA were plated in 12-well plates. Cells were grown on plates to reach confluence (approximately 16 h). Next, a scratch was made in the monolayer using 1 ml pipette tips and pictures were taken at 0 and 12 h. The cell migration (as measured by scratch recovery) was calculated using the following equation (1-T_t_/T_0_) × 100, were T_t_ is the area at 12 h and T_0_ is the area at time zero.

### Ki67 staining

To detect ki67 positive cells in xenograft sections, immunohistochemistry was performed. Briefly, xenograft frozen sections were washed by PBS, incubated in 1% Triton X-100 for 15 min and then blocked in 1% BSA for 30 min. Primary antibodies against ki67 (Abcam, 1:100 dilution) was added and the slides were incubated overnight at 4°C. Slides were washed three times in PBS for 10min. Secondary antibodies were added on to the slides and further incubated for another 1 hour. After 3 times washing in PBS for 10 min. each, slides were stained using DAB kit (Life Technology, Carlsbad, CA) to display the ki67 positive cells stained in brown color.

### Foci (colony) formation assay

Colony formation assay was performed as explained previously [56]. Briefly, equal numbers of PC3, DU145 and LNCaP cells expressing control, GSK3α or GSK3β ShRNA were plated on 6-well plates and incubated in the presence of 10% FBS. After 10 days (20 days in case of LNCaP) under culture conditions, cells were stained with 5% methylene blue (Sigma, St. Louis, MO) in 50% ethanol for 10 min. Colonies of >50 cells were counted.

### Terminal deoxynucleotidyl transferase-mediated dUTP nick end labeling (TUNEL) assay

TUNEL assay for the *in situ* detection of apoptosis was performed using the ApopTag® Red In Situ Apoptosis detection kit (Millipore, MA) according to the manufacturer's instructions. Frozen nude mouse prostate tumor (PC3) xenograft sections were also processed accordingly. Nuclei were counterstained with DAPI. Tissue sections were analyzed for apoptotic cells with localized fluorescence using an inverted fluorescence microscope (Zeiss Axiovert100M, Carl Zeiss, Germany).

### Immunocytochemistry fluorescence staining

For immunofluorescence staining of prostate cancer cells, PC3 and DU145 cells were seeded on to the cell culture chambers, washed two times with PBS, then fixed cells using 4% paraformaldehyde for 20 min. Cells were then incubated with 0.1 % Triton X-100 for 15 min. Cell sections were blocked by 1% BSA, incubated with β-catenin antibodies (1:100) at 4 °C overnight. Immunofluorescence was revealed using Alexa-594 anti-mouse secondary antibodies (1:2000). Cells sections were mounted in fluorescence mounting medium (Vectashield, Vector Laboratories, Burlingame, CA). Samples were observed under a confocal microscope equipped with argon and helio/neon lasers (LSM510, Germany). Controls were performed by omitting either one or both primary antibodies.

### Western blot analysis

Prostate cancer cells were washed twice with PBS and protein was extracted using RIPA buffer (containing 1% Triton × 100, 150 mM NaCl and 50mM Tris buffer, pH 7.4) containing protease and phosphatase inhibitor cocktails (Roche). Proteins were separated by 8-12 % SDS-PAGE gel, and then transferred onto PVDF membrane as described previously [57]. Blotted membranes were blocked with 5 % milk for regular proteins or 5 % BSA for phosphorylated antibodies, and densitometry data as measured using NIH Image J software was normalized with β-actin loading control (Sigma, St Louis, MO).

### *In vivo* nude mouse tumor xenograft model

Stable transfected control, GSK3α or GSK3β ShRNA PC3 cells were grown to confluence in 75-ml flasks. Cells were re-suspended in PBS, counted and equal number of cells (1 × 10^6^) was injected subcutaneously in 7- to 8-week-old nude mice (athymic nude mice; Harlan, Indianapolis, IN). Mice were sacrificed on day 16, and tumors were dissected, weighed, and tissues were fixed in 4% PFA for immunohistochemistry analysis.

### *In vivo* mouse model of prostate cancer colonization to lungs

Eight-week old nude mice (athymic nude mice; Harlan, Indianapolis, IN) were divided into three groups for the administration of PC3 cells expressing control, GSK3α or GSK3β ShRNA. Cells (0.5×10^6^) suspended in normal saline were injected to each group of mice separately via tail vein. In all the experiments mice were evaluated for the presence of metastases 16 days after cell administration. 1.5 ml of 15% India-ink solution was injected intratracheally to stain the lungs and visualize non-stained the tumor nodules. Stained lungs were carefully resected and rinsed in Fekete's solution (300 mL 70% ethanol, 30 mL 37% formaldehyde, 5 mL glacial acetic acid), then placed in fresh Fekete's solution overnight. The whole lungs were harvested for the counting of tumor nodules. Following this, mice lungs were subjected for equal interval cross section (2mm) analysis for PC3 cell colonization to the lung tissues. Tumor area was measured using the NIH Image J software.

### Statistical analysis

All the data are presented as Mean + SD and were calculated from multiple independent experiments. The Student's two-tailed *t* test or ANOVA were used to determine significant differences between treatment and control values using the GraphPad Prism 4.03 software. Data with *p* value < 0.05 was considered significant, and are marked with symbols wherever data are statistically significant.
